# Genetic basis of cefiderocol resistance in *Acinetobacter baumannii*: insights from functional genomics and clinical isolates

**DOI:** 10.1128/spectrum.03804-25

**Published:** 2026-02-09

**Authors:** Kevin J. Rome, José R. Mediavilla, Austin J. Terlecky, Mia J. Bucich, Elena Shashkina, Jianping Jiang, Dillon Kunkle, Albert Rojtman, Eric Skaar, Liang Chen, Barry N. Kreiswith

**Affiliations:** 1 Center for Discovery and Innovation, Hackensack Meridian Health 721734, Nutley, New Jersey, USA; 2 Vanderbilt Institute for Infection, Immunology, and Inflammation, Vanderbilt University Medical Center 12328, Nashville, Tennessee, USA; 3 School of Pharmacy and Pharmaceutical Sciences, University at Buffalo 12292 , Buffalo, New York, USA; University of Pittsburgh, Pittsburgh, Pennsylvania, USA

**Keywords:** carbapenem-resistant *Acinetobacter baumannii*, cefiderocol, transposon mutagenesis, whole-genome sequencing, comparative genomics, antimicrobial resistance, iron metabolism

## Abstract

Cefiderocol (CFDC) is a siderophore-conjugated cephalosporin that hijacks bacterial iron uptake pathways to traverse the outer membrane, offering potent activity against carbapenem-resistant *Acinetobacter baumannii* (CRAB). Although mutations in *pirA*, a TonB-dependent siderophore receptor, have been linked to CFDC resistance, the broader genetic basis remains poorly defined. Using Himar1 transposon mutagenesis in a CFDC-susceptible sequence type 2 (ST2) CRAB strain, we identified ten genes whose disruption reduced CFDC susceptibility, spanning siderophore-mediated uptake (*pirA* and *puiA*), oxidative and redox stress responses (*oxyR*, *nfuA*, *aarF*, *cyoA*, and *bfmRS*), and cell envelope morphogenesis (*mreB*). Most mutants retained wild-type susceptibility to other β-lactams, indicating that reduced CFDC susceptibility can arise independently of target modification. Quantification of cellular iron revealed modest reductions in several mutants, with the largest decreases observed in strains with disruptions in TonB-dependent receptors. Inactivation of *pirA* or *puiA* altered the expression of several alternative TonB-dependent siderophore receptors. Whole-genome sequencing of ST2 clinical isolates with reduced CFDC susceptibility uncovered mutations in TonB-dependent receptors, porins, and PBP3, along with increased β-lactamase expression. Importantly, the β-lactamase inhibitor avibactam restored CFDC susceptibility in isolates with β-lactamase upregulation and intact uptake pathways, whereas strains with concurrent uptake defects remained resistant, underscoring the interplay between permeability and enzymatic drug inactivation. These findings define a multifactorial resistance landscape integrating impaired uptake, redox and envelope stress adaptation, and β-lactamase-mediated drug inactivation.

**IMPORTANCE:**

Cefiderocol (CFDC) is one of the few remaining antibiotics with activity against carbapenem-resistant *Acinetobacter baumannii* (CRAB), an urgent global health threat. Yet, resistance to CFDC is increasingly reported, and the underlying mechanisms remain incompletely defined. Most prior studies have examined single pathways, such as loss of TonB-dependent receptors. Here, we used genome-wide transposon mutagenesis together with genomic and phenotypic analysis of CFDC-resistant clinical isolates to generate a more comprehensive view of how resistance emerges. Our findings show that CFDC resistance is multifactorial, involving disrupted siderophore uptake, alterations in oxidative and envelope-stress responses, porin and cell-wall changes, and β-lactamase activity. By defining how these pathways converge, this work provides a broader mechanistic framework for interpreting emerging resistance in clinical settings. These insights underscore the need for integrated surveillance strategies and highlight the biological complexity that must be considered to preserve the effectiveness of this last-line antibiotic.

## INTRODUCTION

Antimicrobial resistance remains one of the most significant global health challenges, with carbapenem-resistant *Acinetobacter baumannii* (CRAB) recognized as a critical threat by the World Health Organization (1) and the Centers for Disease Control and Prevention (2). A significant proportion of CRAB infections worldwide are caused by sequence type 2 (ST2) strains, also classified as international clone II. ST2 is the most prevalent lineage globally, responsible for major outbreaks across Europe, Asia, and the Americas (3). ST2 strains frequently harbor carbapenemases such as OXA-23 and NDM-1, severely limiting treatment options (4, 5).

The recent introduction of cefiderocol (CFDC), a novel siderophore-conjugated cephalosporin, represents a significant advance in the treatment of Gram-negative infections, including CRAB (6, 7). CFDC is designed to exploit bacterial iron uptake systems, allowing active transport of the drug across the outer membrane via TonB-dependent siderophore receptors (8–10). Once inside the periplasm, CFDC exerts its bactericidal effect by inhibiting penicillin-binding proteins (PBPs), similar to other β-lactams (9). The mechanism of CFDC uptake and activity was initially anticipated to limit the emergence of resistance; however, clinical and *in vitro* studies have already reported CFDC-resistant *A. baumannii* strains (6, 11–13).

Resistance to CFDC is known to involve mutations in TonB-dependent siderophore receptors such as *pirA* and *puiA*, leading to premature stop codons or alterations in essential residues, as well as mutations that result in transcriptional downregulation of these receptors (12–17). However, given the redundancy of these receptors in *A. baumannii*, it remains unclear how loss of a single receptor confers increased resistance. Moreover, other potential mechanisms of CFDC resistance remain poorly characterized, with growing evidence suggesting that factors beyond TonB-dependent uptake contribute to reduced susceptibility (18–20).

Most prior studies investigating CFDC resistance have relied on the selection of spontaneous mutants or the analysis of resistant clinical isolates, which tend to highlight high-frequency events such as mutations in TonB-dependent receptors (10, 21, 22). While these approaches have revealed key uptake mechanisms, they inherently overlook loci whose disruption confers subtler phenotypes or lower-frequency resistance events. To overcome these limitations, we used a CFDC-susceptible ST2 background and performed genome-wide Himar1 transposon mutagenesis to systematically identify genes modulating CFDC susceptibility beyond canonical TonB systems. This forward-genetic approach provided an unbiased framework to uncover both known and previously unrecognized determinants contributing to CFDC susceptibility. Whole-genome sequencing (WGS) of CFDC-resistant clinical ST2 isolates confirmed the relevance of these findings in naturally evolved strains and revealed additional adaptive routes contributing to resistance. Together, these results define a multifactorial resistance landscape involving impaired uptake, stress adaptation, and β-lactamase activity in CRAB.

## MATERIALS AND METHODS

### Bacterial strains and growth conditions

All bacterial strains and plasmids used in this study are listed in Table S1. *A. baumannii* strains were routinely cultured in Luria-Bertani (LB) broth or on LB agar plates at 37°C. For CFDC susceptibility testing, strains were grown on cation-adjusted Mueller-Hinton agar or in iron-depleted cation-adjusted Mueller-Hinton broth (IDMH), as specified (23–25). IDMH was prepared by treating CAMHB with Chelex-100 resin (Bio-Rad) for 6 h to remove divalent cations, followed by re-supplementation with Ca²^+^, Mg²^+^, and Zn²^+^ to the concentrations recommended by the Clinical and Laboratory Standards Institute (CLSI) (25). All *A. baumannii* strains were derived from the clinical isolate BK72560. *Escherichia coli* K-12 strain WM3064 was used for conjugative plasmid transfer into *A. baumannii*. Antibiotic selection was used as follows: apramycin at 30 µg/mL for *E. coli* and 100 µg/mL for *A. baumannii*; rifampin at 100 µg/mL for *E. coli* and 50 µg/mL for *A. baumannii*.

### Antibiotic susceptibility testing

Minimum inhibitory concentrations (MICs) were determined using the broth microdilution method according to CLSI guidelines. For each assay, overnight bacterial cultures were diluted to approximately 5 × 10^5^ CFU/mL in the appropriate medium and incubated with serial twofold dilutions of the antibiotic in 96-well microtiter plates at 37°C for 16–20 h. To investigate the effect of β-lactamase inhibitors on the activity of CFDC against these isolates, MICs were determined with and without 16 mg/L avibactam (AVI), sulbactam (SUL), or tazobactam (TAZ), respectively. Reported MIC values were reproducible across a minimum of three independent biological replicates.

### Transposon insertion mutagenesis

A transposon insertion library was generated in *A. baumannii* BK72560, a CFDC-susceptible ST2 strain, using plasmid pSAMtac1, following the procedure previously described (26). This plasmid harbors a *himar1C9* transposon flanked by MmeI-modified inverted repeats and an apramycin resistance gene marker, along with a rifampin resistance gene (*arr*), on the plasmid backbone. The pSAMtac1 plasmid was introduced into *A. baumannii* BK72560 using conjugation with *E. coli* WM3064 as the donor strain (BK66465). Donor strains were cultured overnight in LB broth supplemented with rifampin (100 µg/mL) and 0.3 mM diaminopimelic acid (DAP). The recipient strain was cultured overnight in LB broth without antibiotics. Donor and recipient cultures were adjusted to an OD_600_ of 0.6–0.8, mixed at a 1:3 donor-to-recipient ratio, pelleted by centrifugation at 4,500 × *g* for 10 min, washed, and resuspended in fresh LB broth containing 0.3 mM DAP and 100 µM IPTG. The mixture was spotted onto non-selective LB + DAP agar plates and incubated at 37°C for 16 h. Bacterial lawns were scraped, resuspended in 1 mL of LB, serially diluted, and plated onto LB agar supplemented with apramycin (100 µg/mL). Plates were incubated at 37°C for 24 h, and apramycin-resistant colonies were pooled to create a transposon mutant library.

### Deep sequencing of Tn insertions and analysis of sequencing data

The Tn mutant DNA library was sequenced on an Illumina HiSeq platform, generating 10.4 million 150-bp paired-end reads. Reads were quality- and adapter-trimmed using Trimmomatic v0.39 (27) and processed with the Tn-Seq PreProcessor (TPP) (27), which counts reads mapping to each TA dinucleotide site in the *A. baumannii* BK72560 genome. Output wig files from TPP were used to calculate library saturation and insertion density (insertions per kilobase), to assess library diversity.

### Screening the transposon library for reduced CFDC susceptibility

An aliquot of the *A. baumannii* BK72560 transposon library was grown in Mueller-Hinton (MH) broth at 37°C with shaking until the culture reached an OD_600_ of 0.1. A 100 µL aliquot of the culture (1 × 10^7^ CFU) was spread onto MH agar plates supplemented with CFDC (2 µg/mL) and incubated at 37°C for 24 h. Resistant colonies were patched onto LB agar supplemented with apramycin (100 µg/mL), and MH agar supplemented with CFDC (2 µg/mL). Colonies that grew on both selective media were retained for further analysis. The location of the transposon insertion in confirmed mutants with reduced CFDC susceptibility was determined by arbitrary PCR using primers listed in Table S2 (28). PCR products were sequenced, and the insertion sites were mapped to the *A. baumannii* BK72560 genome.

### Construction of the pKR-AB expression plasmid

Standard techniques were used for DNA manipulation. Plasmid backbone amplification of pMApra-Para (29) was performed using the primers pMA-ara-F (5′-CTGACGATCGAAACGTGGCCAATATGGACA-3′) and pMA-ara-R (5′-TGACGCGGCCGCTTGGTAACGAATCAGACAATTGAC-3′), introducing PvuI and NotI restriction sites. The *rifR* gene, encoding rifampin resistance, was amplified from plasmid pSGKp (30) using the primers rifR-F (5′-ACGTGCGGCCGCGGAACCCCTATTTGTTTATTTTTC-3′) and rifR-R (5′-ACGTCGATCGAACTTGGTCTGACAGCTAGT-3′), introducing the same PvuI and NotI restriction sites, respectively. PCR amplification was carried out using Q5 High-Fidelity DNA Polymerase (New England Biolabs). Both the *rifR* fragment and ∆*apmR* pMA backbone were digested with PvuI and NotI restriction enzymes (New England Biolabs), and DNA ligation was performed using T4 DNA Ligase (New England Biolabs). The recombinant pKR-AB1 plasmid was introduced into chemically competent DH5α *E. coli* cells (New England Biolabs) using the standard heat-shock transformation protocol. Transformants were selected on LB agar plates supplemented with 100 μg/mL rifampin (Sigma-Aldrich). Positive clones were confirmed by restriction digestion analysis and Sanger sequencing. For genetic complementation assays, each ORF of the gene of interest, along with its native ribosome binding site, was cloned under the control of the *araB*p promoter in the pKR-AB1 vector. The primers used for cloning are listed in Table S2. For transformation into *A. baumannii*, electrocompetent cells were used, and transformants were selected on LB plates supplemented with 50 μg/mL rifampin (Sigma-Aldrich). Gene expression was induced with 2% (wt/vol) l-arabinose (Sigma-Aldrich).

### RNA isolation

Total bacterial RNA was isolated from *A. baumannii* grown in IDMH, with or without 1× MIC CFDC. RNA was harvested after 5 h of incubation at 37°C, following the time point established in previous studies as yielding maximal CFDC-responsive expression of TonB-dependent receptors (31). RNA extraction was performed using the RNeasy Plus Mini Kit (Qiagen) according to the manufacturer’s instructions. Remaining traces of DNA in the isolated RNA samples were eliminated by a 30-min treatment with Turbo DNA-free DNase (ThermoFisher) as described by the manufacturer. NanoDrop 1000 Spectrophotometer (Thermo Scientific, USA) was used for quantification of RNA and purity determination. The absence of contaminating genomic DNA was confirmed by failure to amplify the housekeeping gene *rpoD* by PCR.

### Reverse transcription-quantitative PCR (qRT-PCR)

qRT-PCR was performed in an AriaMx real-time PCR system (Agilent Technologies, Santa Clara, CA, USA) using a Brilliant II SYBR Green qRT-PCR 1-Step kit (Agilent). Following the manufacturer’s instructions, reactions were carried out in a final volume of 20 μL with 50 ng RNA and 1 μL of each primer at 5 µM. Reverse transcription was performed by incubating at 50°C for 55 min followed by PCR amplification (95°C for 10 min, 35 cycles of 30 s at 95°C, 30 s at 55°C, and 30 s at 72°C, and 72°C for 1 min). The relative abundances of target and reference gene (Table S3) mRNA were quantified in triplicate. Comparisons between gene expression levels in media with versus without CFDC were determined using Student’s *t*-test.

### Inductively coupled plasma mass spectrometry (ICP-MS)

Three biological replicates of *A. baumannii* strains were grown in 5  mL of MH broth at 37 °C for 5 h with shaking. Cultures were harvested by centrifugation and washed with PBS. Cell pellets were digested overnight in 70% trace-metal–grade nitric acid at 65 °C and subsequently diluted to 20% nitric acid using ultrapure water. Elemental quantification (Fe, Mn, Zn, and Cu) was performed using an Agilent 7700 ICP-MS coupled to an ASX-560 autosampler. Samples were introduced via a peristaltic pump through 0.5 mm internal-diameter tubing and a MicroMist borosilicate glass nebulizer. Uptake was carried out at 0.5 rps for 30 s, followed by 30 s at 0.1 rps to stabilize the signal. Spectrum mode analysis was conducted at 0.1 rps, with three points collected across each peak and three replicates of 100 sweeps per element. Instrument settings included: cell entrance = −40V, cell exit = −60V, plate bias = −60V, OctP bias = −18 V, and helium flow = 4.5  mL/min. Voltages for extract 2, omega bias, omega lens, OctP RF, and deflect were empirically optimized. Calibration curves were generated using ARISTAR ICP standard mixes. Between each sample, the sampling probe and tubing were rinsed with 2% nitric acid for 30 s at 0.5 rps. Final metal concentrations were normalized to ^34^S to account for variations in biomass. Data acquisition and analysis were performed using Agilent MassHunter Workstation software (version A.01.02).

### WGS

All *A. baumannii* clinical isolates were submitted to WGS as follows. DNA extraction was performed using WizardGenomic DNA Purification Kit from Promega (Promega Corporation, WI, USA). All isolates underwent short-read WGS using the Illumina HiSeq 2500 platform with 2 × 150 bp paired-end reads (Illumina, Inc, CA, USA). Illumina sequences were trimmed using Trimmomatic v0.39 (27), and assembled *de novo* using SPAdes v4.0.0. (32). Identification of resistance genes was performed using AMRFinderPlus v4.0.23 (33) with the assembled genomes. Using *A. baumannii* strain AB5075-UW (NCBI: NZ_CP008706.1) as a reference sequence, Snippy v4.6.0 (34) was used with the Illumina sequence reads to identify mutations among these clinical isolates.

## RESULTS

### Transposon mutagenesis in β-lactam-resistant *A. baumannii* BK72560

**Fig 1:**
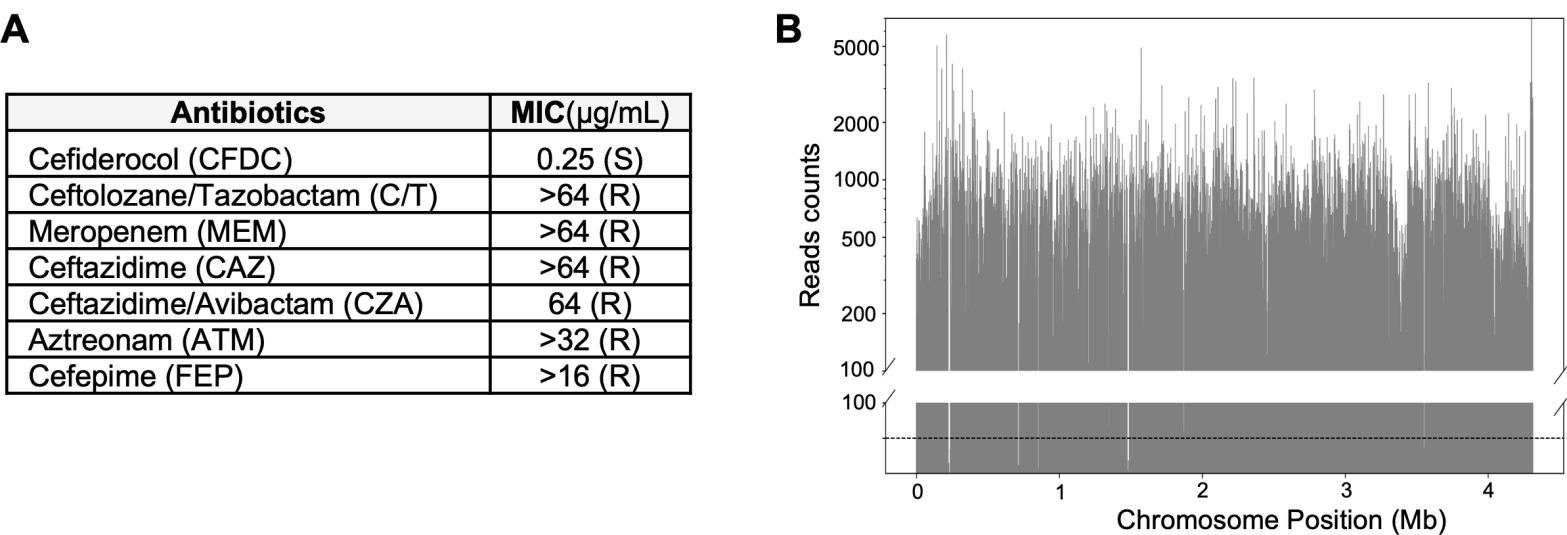
Antibiotic susceptibility profile and transposon library coverage for *A. baumannii* BK72560. (**A**) MIC values for β-lactam antibiotics (25). breakpoints were used to define susceptibility (S) or resistance (R). (**B**) Genome-wide distribution of transposon insertion read counts across the ~4.2 Mb chromosome. Dashed line marks the ≥50 insertions/gene threshold.

To identify genetic determinants contributing to both high-level and low-level reductions in CFDC susceptibility, we employed a genome-wide transposon mutagenesis approach to comprehensively map genes influencing CFDC susceptibility in a clinical CRAB strain. We selected *A. baumannii* BK72560, a sequence type ST2 isolate recovered from a bloodstream infection in the United States. Genomic analysis revealed the presence of several β-lactamase genes, including *bla*_OXA-66_, *bla*_OXA-23_, and *bla*_ADC-25_, contributing to its extensive resistance profile to carbapenems, cephalosporins, and monobactams (Fig. 1A). Despite this β-lactam resistance, BK72560 remains susceptible to CFDC, making it a suitable background for transposon mutagenesis to identify loci associated with reduced CFDC susceptibility.

We therefore constructed a HimarC1 transposon insertion mutant library in BK72560. Illumina-based transposon sequencing (Tn-seq) revealed a median insertion saturation of 24% across “TA” dinucleotide sites. Despite this moderate saturation, the library exhibited broad genome coverage, with a non-zero mean (NZ_mean) of approximately 122 insertions per gene, and an average of 17 insertions per kb, consistent with thresholds generally considered sufficient for genome-wide fitness profiling (NZ_mean ≥ 50) (Fig. 1B ) (34).

### Identification of genetic determinants of reduced CFDC susceptibility

**Fig 2:**
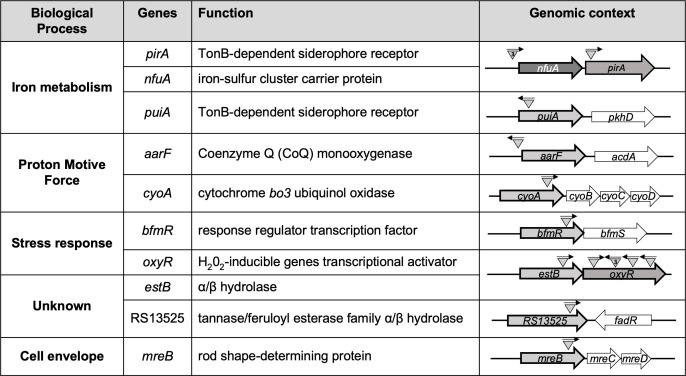
Transposon-disrupted genes associated with reduced CFDC susceptibility. Disrupted genes, predicted functions, and biological processes were identified in mutants with decreased CFDC susceptibility. Genomic context is shown with transposon insertions marked as gray triangles; arrowheads indicate insertion orientation. Numbers within triangles denote independent isolates with insertions at the same site. Disrupted genes are in gray with black outline; neighboring genes are shown for reference.

To select mutants with reduced susceptibility to CFDC, the transposon library was plated on MH agar containing 2 µg/mL CFDC, which corresponds to 8× MIC of the parental strain (0.25 µg/mL). Seventeen transposon mutants exhibiting growth under these conditions were recovered. The reduced-susceptibility phenotype of the selected mutants was confirmed by patching onto MH agar containing 2 µg/mL CFDC. Transposon insertion sites were determined using arbitrary PCR. The disrupted genes associated with reduced susceptibility to CFDC, along with their genomic contexts, including adjacent genes and predicted operon structures, are summarized in Fig. 2.

We identified transposon insertions in 10 unique genes of *A. baumannii*. Two insertions were found in genes encoding TonB-dependent siderophore receptors including RS16540 (*puiA*) and RS14160 (*pirA*). Three independent colonies contained sibling transposon insertions located 20 bp upstream of RS14165 (*nfuA*), which lies directly upstream of *pirA* (35). Insertions were also identified in RS15450 (*bfmR*), the regulator of the two-component system BfmRS, and in both genes of the RS14100 (*estB*)-RS14105 (*oxyR*) operon. Six independent colonies contained transposon insertions within *oxyR*, distributed across four distinct sites, including one position represented by three sibling insertions occurring at the same locus. The *estB* gene encodes an alpha-beta hydrolase, while *oxyR* is involved in oxidative stress response. Additional disrupted genes included RS07580 (*cyoA*), encoding cytochrome *bo_3_* ubiquinol oxidase*;* RS07705 (*aarF*)*,* required for ubiquinone biosynthesis; RS03530 (*mreB*), encoding a rod shape-determining protein; and RS13525, encoding a tannase/feruloyl esterase. WGS confirmed that the selected mutants were otherwise isogenic to the parental BK72560 strain, supporting that the observed phenotypes are attributable to the transposon insertions.

### β-lactam susceptibility profiles of transposon mutants with reduced CFDC susceptibility

**Fig 3:**
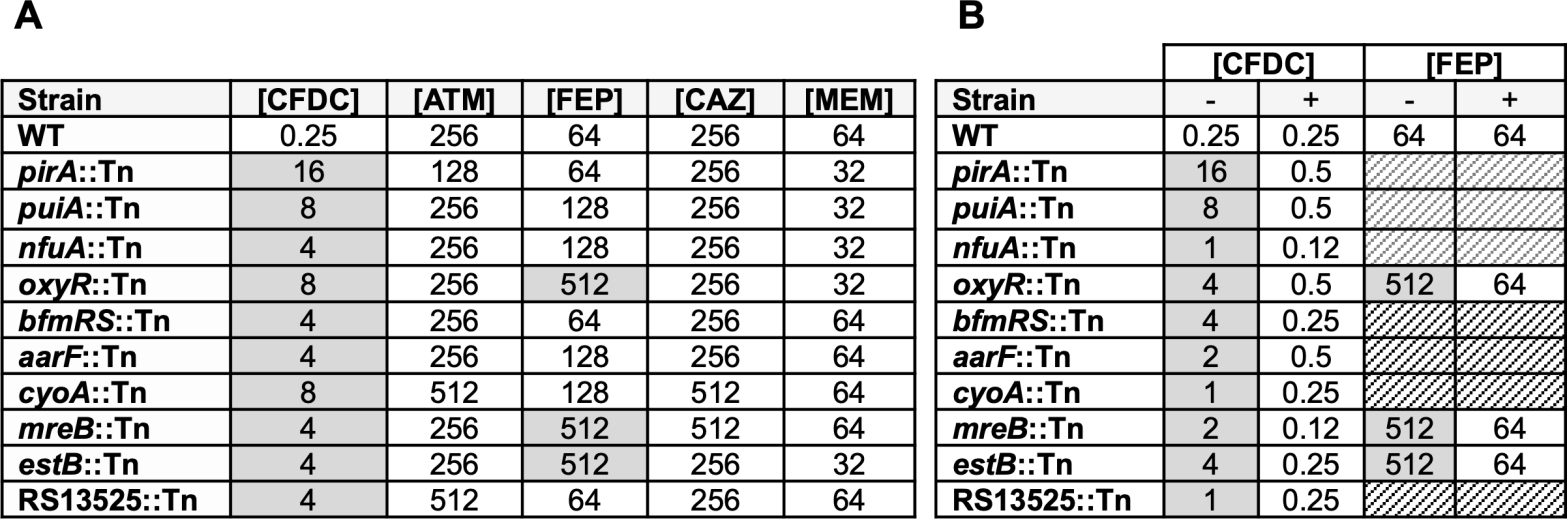
Susceptibility of CFDC-resistant *A. baumannii* BK72560 transposon mutants and complemented strains to β-lactam antibiotics. (**A**) MICs (µg/mL) for the wild-type (WT) BK72560 strain and transposon insertion mutants recovered following selection on MH agar containing 2 µg/mL CFDC. Each mutant harbors a single transposon insertion in the indicated gene. MICs were determined by broth microdilution. Shaded cells indicate a ≥4-fold change relative to the WT. Antibiotics tested: CFDC, ceftazidime (CAZ), aztreonam (ATM), cefepime (FEP), and meropenem (MEM). (**B**) Complementation analysis of CFDC and FEP susceptibility. Mutants were transformed with either an empty vector (–) or a plasmid expressing the corresponding WT gene in *trans* under the control of the arabinose-inducible promoter (*araB*p) (+). MICs were determined by broth microdilution in media supplemented with 2% arabinose. Shaded cells indicate a ≥4-fold change relative to the WT. Striped cells indicate that the condition was not tested.

The genes identified in our screen span diverse functional categories, including iron acquisition, oxidative stress response, respiration, cell envelope integrity, and periplasmic hydrolase activity. To assess whether these mutations also impact susceptibility to other β-lactams, we measured MICs of the transposon insertion mutants and wild-type BK72560 against a panel of β-lactam antibiotics (Fig. 3A). Most transposon mutants isolated on CFDC (2 µg/mL) exhibited elevated CFDC MICs compared to the parental strain. Notably, the *pirA*::Tn showed a 64-fold increase in CFDC MIC without changes to other β-lactams, indicating a CFDC-specific effect. Similar increases were observed for *puiA::Tn* (32-fold), *bfmRS::Tn* (16-fold), and RS13525::Tn (16-fold), reinforcing their potential roles in mediating CFDC susceptibility. Notably, *estB::Tn*, *oxyR::Tn*, and *mreB::Tn* mutants exhibited decreased susceptibility to both CFDC and cefepime.

To confirm that the identified genes were responsible for the reduced-susceptibility phenotype, we performed complementation assays by introducing the native gene in *trans* (Fig. 3B). Complementation of *pirA::Tn*, *puiA::Tn*, *bfmRS::Tn*, and *nfuA::Tn* restored susceptibility to CFDC. Complementation of *estB::Tn*, *oxyR::Tn*, and *mreB::Tn* restored susceptibility to both CFDC and cefepime, supporting their broader role in β-lactam resistance.

### Metal content profiles in mutants with reduced CFDC susceptibility

**Fig 4:**
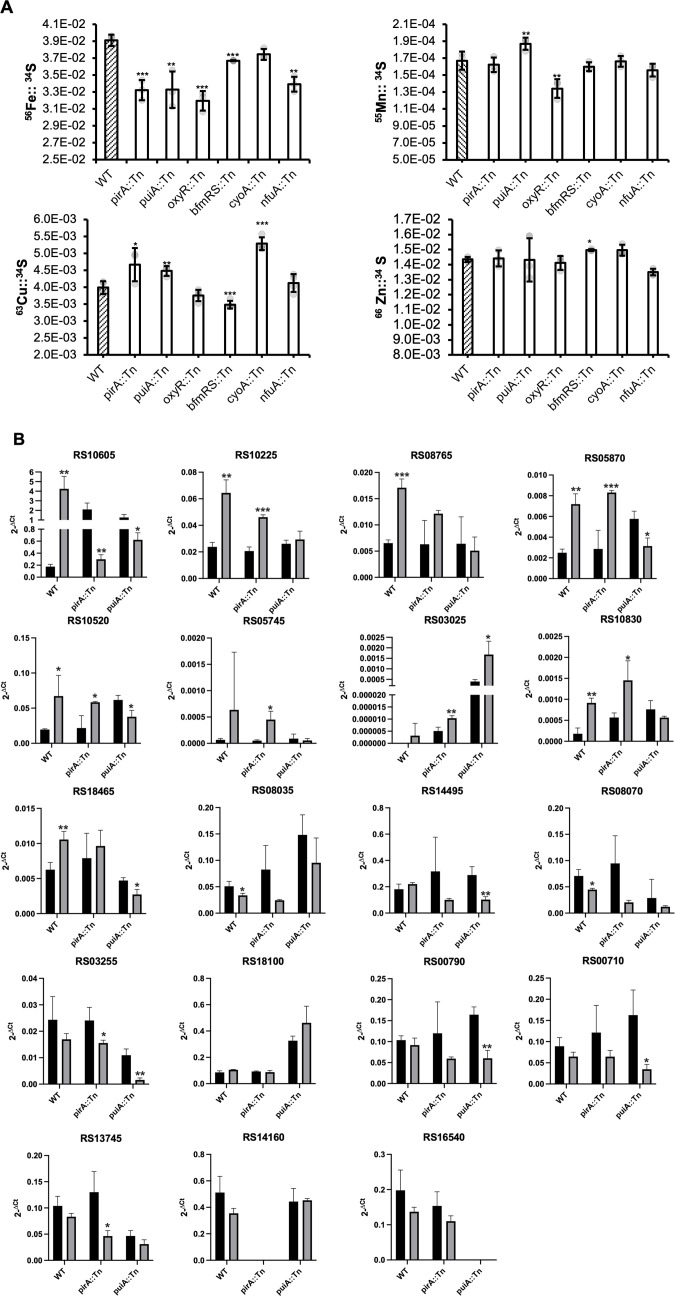
(**A**) ICP-MS-based profiling of Fe, Mn, Cu, and Zn content in *A. baumannii* BK72560 and transposon mutants derivatives. Metal isotopes ^56^Fe, ^55^Mn, ^63^Cu, and ^66^Zn were measured by ICP-MS in *A. baumannii* grown in Mueller-Hinton for 5 h. Data are mean ± standard deviation normalized to ^34^S to account for differences in growth of biological replicates. Statistical significance was determined by *t*-test, comparing each transposon mutant to the parental strain. Significance thresholds were defined ns = *P* > 0.05, * = *P* < 0.05, ** = *P* < 0.01, *** = *P* < 0.001, and **** = *P* < 0.0001. (**B**) Fold changes in expression levels of TonB-dependent siderophore receptors in the presence of 1× MIC CFDC relative to its absence. qRT-PCR was performed on WT strain, *pirA*::Tn, and *puiA*::Tn grown in IDMH with or without 1× CFDC MIC. Target gene levels were normalized to the housekeeping gene *rpoD*, expression is reported using the 2^−ΔC*t*^ method. Bars represent the mean of biological replicates, with error bars indicating standard deviation. Statistical significance was assessed using a two-tailed *t*-test, comparing each isolate to IDMH. Significance thresholds: ns = *P* > 0.05, * = *P* < 0.05, ** = *P* < 0.01, *** = *P* < 0.001, and **** = *P* < 0.0001.

Most mutants with reduced CFDC susceptibility had β-lactam MICs similar to the parental strain, arguing against changes in β-lactam targets and raising the possibility of altered uptake. We therefore quantified cellular metal content by ICP-MS in selected mutants grown in iron-replete media to determine whether they exhibited detectable shifts in metal homeostasis (Fig. 4A). Elemental abundances were normalized to cellular sulfur (34^S^) content to account for differences in cellular biomass. Growth kinetics of each mutant were also compared to the parental strain (Fig. S1), and all mutants exhibited similar growth profiles. All mutants tested exhibited reduced cellular iron compared to BK72560. The most pronounced reductions were observed in *oxyR*::Tn (18%), *pirA*::Tn (15%), *puiA*::Tn (14%), and *nfuA*::Tn (13%), while slight decreases were also seen in *bfmRS::*Tn (6%) and *cyoA*::Tn (4%). Beyond iron, distinct patterns emerged for other metals: *oxyR*::Tn also exhibited decreased manganese and copper, whereas *pirA*::Tn, *puiA*::Tn, *cyoA*::Tn accumulated more copper. *bfmRS*::Tn displayed reduced copper alongside elevated zinc, while *nfuA*::Tn showed a modest reduction in zinc. Cellular iron content appeared reduced in most transposon mutants, with the strongest decreases observed in *oxyR::Tn*, *pirA::Tn*, and *puiA::Tn*.

Given that *pirA* and *puiA* encode TonB-dependent siderophore receptors involved in CFDC uptake, we measured the transcriptional response of 18 TonB-dependent receptors in the WT strain as well as the *pirA::*Tn and *puiA::*Tn mutants grown under iron-depleted conditions with or without CFDC exposure (Fig. 4B). In the WT background, CFDC exposure induced a defined subset of receptors including RS10605, RS10225, RS08765, RS0575, RS05870, RS10520, RS10850, and RS18465, while the remaining loci showed little or no change. In the *pirA::*Tn mutant, the CFDC-responsive pattern observed in the WT strain was altered. Several receptors that were induced in the WT strain after CFDC exposure, including RS10605, RS08765, and RS18465, were not upregulated. RS10605 showed elevated expression under iron-depleted conditions relative to WT, and its transcript abundance was lower in the presence of CFDC. RS13745 also showed reduced expression following CFDC treatment, and RS03025 expression was markedly higher than in the WT strain. The *puiA::*Tn mutant exhibited a partially similar profile with reduced induction of RS10605 after CFDC exposure and increased expression of RS03025, along with lower transcript levels for several receptors in the presence of CFDC. Together, these findings indicate that loss of a single TonB-dependent receptor alters the transcriptional profile of alternative receptors and the response to CFDC.

### Genomic profiling of ST2 clinical isolates highlights the multifactorial basis of CFDC resistance

Our data suggest that CFDC resistance in *A. baumannii* involves multiple determinants, with TonB-dependent siderophore receptors emerging as key contributors. To further investigate resistance mechanisms, we performed WGS and susceptibility profiling on eight ST2 CRAB clinical isolates. MICs were determined in the presence and absence of the β-lactamase inhibitors AVI, SUL, and TAZ to distinguish β-lactamase-mediated effects from alternative mechanisms (Table 1). In the CFDC-susceptible isolates BK72560 and BK45355, inhibitor addition had little or no impact on MICs. The CFDC-intermediate isolate BK51868 similarly showed no reduction in MICs, suggesting non-β-lactamase-mediated resistance. In contrast, AVI substantially decreased MICs in three resistant isolates (BK45311, BK78055, and BK45353), indicating that β-lactamase activity contributes to CFDC resistance in these backgrounds. Notably, SUL MICs were 2 µg/mL in BK78055, BK51868, BK72560, and BK45355, but exceeded 16 µg/mL in BK45311, BK74166, and BK45353, consistent with resistance potentially driven by target modification.

**TABLE 1:** Genomic features of carbapenem-resistant *A. baumannii* isolates in relation to CFDC susceptibility^*a*^

	[CFDC]				β-lactamase	TonB siderophore	Cell envelope
	–	+AVI	+TAZ	+SUL			
BK72560	0.25 (S)	0.12 (S)	0.12 (S)	0.5 (S)	Bla_OXA-66_ Bla_OXA-23_ ADC-212		
BK45355	0.5 (S)	1 (S)	0.5 (S)	0.5 (S)	Bla_OXA-66_ Bla_OXA-23_ ADC-212		
BK51868	8 (I)	8 (I)	8 (I)	8 (I)	Bla_OXA-82_ Bla_OXA-23_ ADC-33	RS00710^Fs 351/729^ RS03255^Fs 656/734^	OprD^*(x9)^
BK74166	32 (R)	16 (R)	32 (R)	32 (R)	Bla_OXA-113_ Bla_OXA-23_ ADC-56	RS18100^Fs 1/706^	PBP1A* AmpD****
BK78055	64 (R)	8 (I)	62 (R)	62 (R)	Bla_OXA-23 x3_ Bla_OXA-82_ ADC-33		
BK78351	128 (R)	256 (R)	128 (R)	128 (R)	Bla_OXA-23_ Bla_OXA-66_ ADC-293	PirA^Ter 159/754^ RS18465^Ter 231/699^ RS18100^Fs 1/706^	AmpD**** PBP3* PBP1A* OprD^Ter 243/448^
BK45311	128 (R)	8 (I)	64 (R)	64 (R)	Bla_OXA-66_ Bla_OXA-23_ Bla_OXA-72 x2_ ADC-30		AmpD**
BK45353	1024 (R)	4 (S)	1024 (R)	1024 (R)	Bla_OXA-66_ Bla_OXA-23_ Bla_TEM-1_ ADC-143		AmpD** PBP1B* PBP3*

^*a*^ CFDC MICs were determined for eight CRAB clinical isolates in the absence (–) and presence (+) of the β-lactamase inhibitors avibactam (AVI), sulbactam (SUL), and tazobactam (TAZ). Susceptibility categories (susceptible [S], intermediate [I], and resistant [R]) were defined according to CLSI (25) breakpoints.Gray shading indicates a ≥4‑fold change in the presence of a β‑lactam inhibitor compared with CFDC treatment alone. Acquired β-lactamase genes and chromosomal ADC variants included in the table were identified using AMRFinderPlus. Disruptive mutations in TonB-dependent siderophore receptors and cell envelope proteins were identified using snippy with the *A. baumannii* AB5075-UW (ST2) genome as reference. Asterisks (*) indicate substitutions affecting key functional residues; “fs” denotes frameshift mutations; “ter” denotes premature stop codons. Mutation positions are reported as X/Y, indicating the affected residue (X) and the total protein length (Y).

To identify the genetic determinants underlying these susceptibility patterns, we analyzed chromosomal variants using Snippy with AB5075-UW as the reference and annotated resistance genes with AMRFinderPlus (Table 1; Table S4). Resistance gene annotation showed that all isolates encoded variants of both *bla*_OXA_ and *bla*_ADC_. All strains carried *bla*_OXA-23_, with BK78055 harboring three copies. Most isolates also carried *bla*_OXA-66_, except BK78055, BK74166, and BK51868. Additional β-lactamases occurred only in CFDC-intermediate or resistant isolates, including *bla*_OXA-82_ in BK51868 and BK78055, duplicated *bla*_OXA-72_ in BK45311, *bla*_OXA-113_ in BK74166, and *bla*_TEM-1_ in BK45353. Because *bla*_OXA-23_ and *bla*_OXA-66_ were present in both susceptible and non-susceptible isolates, their presence alone does not explain differences in CFDC susceptibility, although differences in β-lactamase copy number and allelic variation may contribute to resistance. In addition to allelic and copy-number variation, we observed evidence of regulatory influences on β-lactamase expression in CFDC-resistant isolates. BK45353, BK75351, and BK45311 each carried amidase-domain mutations in *ampD*, the negative regulator of *bla*_ADC_, and all demonstrated increased *bla*_ADC_ transcription (Fig. S2).

Given the established role of TonB-dependent siderophore receptors in CFDC uptake, we examined these genes for disruptive frameshift or truncating mutations. No unequivocal loss-of-function mutations were observed in the CFDC-susceptible isolates BK72560 and BK45355, nor in the CFDC-resistant isolates BK45311, BK45353, and BK78055. In contrast, three isolates carried loss-of-function alleles in TonB-linked receptors. BK51868 (CFDC-intermediate) carried frameshift mutations in RS03255 ^Fs 656/734^ and RS00710 Fs^351/729^. BK78351 (CFDC-resistant) harbored disruptive mutations in RS14160/PirA^Ter 159/754^, RS01800^Fs 1/706^, and RS18465^Ter 231/699^. BK74166 harbored a frameshift in RS01800^Fs 1/706^. Because CFDC transport also depends on porins, we examined porin loci and identified a premature stop codon in OprD^Ter 243/448^ in BK78351 and nine OprD missense mutations in BK51868, including substitutions previously associated with decreased carbapenem susceptibility (31). Finally, mutations in PBPs, which are the cellular targets of β-lactams, were identified in CFDC-resistant strains. PBP3 A515V (*ftsI*/RS01390) was present in BK45353 and BK75351. BK45353 additionally carried PBP1b A49S (*mrcB*/RS06625), while BK78351 and BK74166 harbored PBP1a T244A (*ponA*/RS01420).

Collectively, the genomic and phenotypic data indicate that reduced CFDC susceptibility in ST2 CRAB isolates arises through multiple, strain-specific combinations of mutations affecting drug entry, β-lactamase activity, and cell-wall–associated pathways.

## DISCUSSION

CFDC, a siderophore cephalosporin hybrid, represents a promising therapeutic option against multidrug-resistant *A. baumannii*, including carbapenem-resistant strains (CRAB). However, the emergence of CFDC resistance in clinical settings underscores the need to unravel the genetic underpinnings of reduced susceptibility. While spontaneous mutant selection can identify prevalent resistance mechanisms, it tends to emphasize recurrent mutations in uptake systems such as TonB receptors, thereby missing lower frequency but functionally relevant events. Our genome-wide transposon mutagenesis provided an unbiased framework to uncover previously underappreciated determinants of CFDC resistance. Integrating these findings with WGS of CFDC-resistant clinical isolates provided a comprehensive view of the multifactorial genetic architecture underlying CFDC resistance in ST2 *A. baumannii*.

Our transposon screen identified 10 genes whose disruption conferred reduced CFDC susceptibility, falling broadly into pathways affecting (i) siderophore uptake, (ii) oxidative/redox stress response, and (iii) cell envelope integrity. We recovered insertions in *pirA* and *puiA*, encoding TonB-dependent receptors known to mediate CFDC uptake (11, 12, 14, 16, 18, 19, 36). Despite the presence of other iron acquisition pathways, disruption of either receptor alone elevated CFDC MICs (Fig. 3), highlighting their importance in CFDC susceptibility. To assess the physiological consequences of receptor loss, we measured cellular metal content and expression of TonB-linked receptors. ICP–MS (Fig. 4A) revealed modest but consistent decreases in cellular iron in *pirA::Tn* and *puiA::Tn*, suggesting impaired iron homeostasis, while transcriptional profiling showed altered expression of alternative receptors and a modified CFDC response. Consistent with the importance of TonB-dependent receptors for CFDC susceptibility, several CFDC-resistant clinical isolates also carried disruptive mutations in these receptors. Notably, *pirA* emerged in both our mutagenesis screen and in resistant clinical isolates, underscoring the robustness of the mutagenesis approach in capturing clinically relevant determinants of CFDC resistance. Mutations affecting outer membrane porins, including OprD truncations, were also detected and may further exacerbate permeability defects (7, 31, 37).

Beyond uptake-related mechanisms, our screen also uncovered mutations in genes linked to iron-dependent respiration and redox homeostasis. These included *nfuA*, which encodes an Fe-S cluster carrier required for respiratory function and redox balance (35, 38, 39) as well as *aarF* (ubiquinone biosynthesis), *cyoA* (cytochrome bo₃ oxidase), and *oxyR* (oxidative stress regulator). Disruption of any of these genes increased CFDC MICs (Fig. 3). It is worth noting that several CFDC-resistant clinical isolates also harbored disruptive mutations in redox-linked genes, including PdxR^Ter 444/499^ (BK74166), GabD^Ter 870/2452^ (BK45311), and FghA^Ter 95/282^ (BK45353) (Table S4). Previous studies have shown that CFDC exposure can induce accumulation of NADH, NADPH, FADH₂, and reactive oxygen species, implicating redox imbalance in its bactericidal activity (22, 40, 41). In *E. coli*, deletion of NADH-quinone oxidoreductase (*nuo* operon) significantly increases CFDC MICs without affecting cefepime or ceftazidime, suggesting a specific vulnerability to CFDC-induced oxidative stress (40). In this context, inactivation of *nfuA*, *cyoA*, and *aarF* may attenuate CFDC-induced oxidative stress. Because TonB-coupled siderophore receptors rely on the proton motive force (PMF) (42), inactivation of PMF-supporting genes such as *aarF* or *cyoA* could likewise diminish CFDC uptake, although the slight reduction in cellular iron (Fig. 4A) is unlikely to fully explain the observed reduction in CFDC susceptibility. Our screen also identified an insertion in *bfmR*, a global regulator involved in envelope stress, redox balance, and efflux control (43). Other two-component system regulators have also been implicated in CFDC resistance. Inactivation of BaeSR, for instance, has been shown to upregulate efflux pumps and increase CFDC MICs (44).

Finally, we identified changes in genes influencing cell envelope structure and β-lactam interaction. Insertions in *mreB*, encoding a bacterial actin homolog required for lateral wall synthesis, conferred resistance to CFDC and cefepime (Fig. 3). Loss of *mreB* disrupts peptidoglycan dynamics, has been shown to reduce reliance on PBP3, and is linked to reduced susceptibility to β-lactams (45–48). WGS also revealed additional resistance-associated alterations, including mutations in PBPs, the primary CFDC target in highly resistant isolates consistent with previous reports in *A. baumannii* and *P. aeruginosa* (7, 18). β-Lactamase-associated mechanisms likewise contributed to CFDC resistance among our clinical isolates (Table 1). Resistant strains exhibited diversity in allelic diversity, copy number, and expression levels among the β-lactamases. Recent work in *P. aeruginosa* demonstrated that expression of a diverse panel of acquired β-lactamases (*bla*_OXA_ and *bla*_KPC_) in an isogenic background increases CFDC MICs (49). Similarly, expression of *A. baumannii* and *P. aeruginosa* AmpC variants in isogenic *E. coli* increases CFDC MICs with mutations within the Ω-loop or R2-loop of AmpC raising MICs by 8- to 16-fold (20, 50, 51). Additionally, mutations in the amidase domain of *ampD*, a negative regulator of *bla*_ADC_, have been shown to modestly increase CFDC MICs (52). Although β-lactamases contribute to CFDC resistance, their impact depends strongly on the integrity of CFDC uptake pathways. We observed that AVI restored susceptibility in isolates with intact TonB-dependent receptors and porins, whereas isolates combining elevated β-lactamase activity with mutations in TonB-dependent receptors and/or porins remained resistant in agreement with recent findings (49). This supports the hypothesis that, beyond well-established mechanisms, additional underappreciated pathways may compound CFDC resistance in clinical isolates.

Taken together, our findings indicate that CFDC resistance in *A. baumannii* arises through multiple interacting mechanisms, including impaired uptake, disruption of redox and cell envelope stress pathways, increased β-lactamase activity, and mutations in target PBPs. Our transposon screen successfully captured both established and previously unrecognized contributors to CFDC resistance, while WGS of clinical isolates revealed mutations in essential genes and subtle allelic variants not accessible by insertional mutagenesis. As one of the few new agents active against multidrug-resistant Gram-negative pathogens, the clinical utility of CFDC will depend on continued efforts to monitor and understand resistance. This study offers a framework for understanding and monitoring CFDC resistance in clinical *A. baumannii* populations and may help guide strategies to preserve its efficacy.

## Data Availability

The genome sequences have been deposited in DDBJ/ENA/GenBank under the bioproject PRJNA549322.

## SUPPLEMENTAL MATERIAL

Figure S1 spectrum.03804-25-s0001.tiff Growth kinetics of *A. baumannii* BK72560 and transposon derivatives in cation-adjusted Mueller-Hinton broth (panel MH) or iron-depleted MH (panel IDMH).

Figure S2 spectrum.03804-25-s0002.tiff qRT expression analysis of blaADC in *A. baumannii* clinical isolates carrying ampD mutations. Cultures were grown in IDMH broth. Gene expression was normalized to the housekeeping gene rpoD and calculated using the 2-ΔCt method.

Table S1 spectrum.03804-25-s0003.docx Bacterial strains and plasmids used in this study.

Table S2 spectrum.03804-25-s0004.docx Primers used in this study for arbitrary PCR and cloning.

Table S3 spectrum.03804-25-s0005.docx Primers used for gene expression quantification by qRT-PCR.

Table S4 spectrum.03804-25-s0006.xlsx Non-synonymous variants identified in *A.baumanii* clinical isolates using AB5075-UW (NCBI: NZ_CP008706.1) as the reference genome and Snippy v4.6.0.

ASM does not own the copyrights to Supplemental Material that may be linked to, or accessed through, an article. The authors have granted ASM a non-exclusive, world-wide license to publish the Supplemental Material files. Please contact the corresponding author directly for reuse.
