# Supplementary figures and images for: Genetic basis of cefiderocol resistance in Acinetobacter baumannii: insights from functional genomics and clinical isolates
Source: Microbiol Spectr. 2026 Feb 9;14(3):e03804-25. doi: 10.1128/spectrum.03804-25 (PMC12955420; doi:10.1128/spectrum.03804-25)

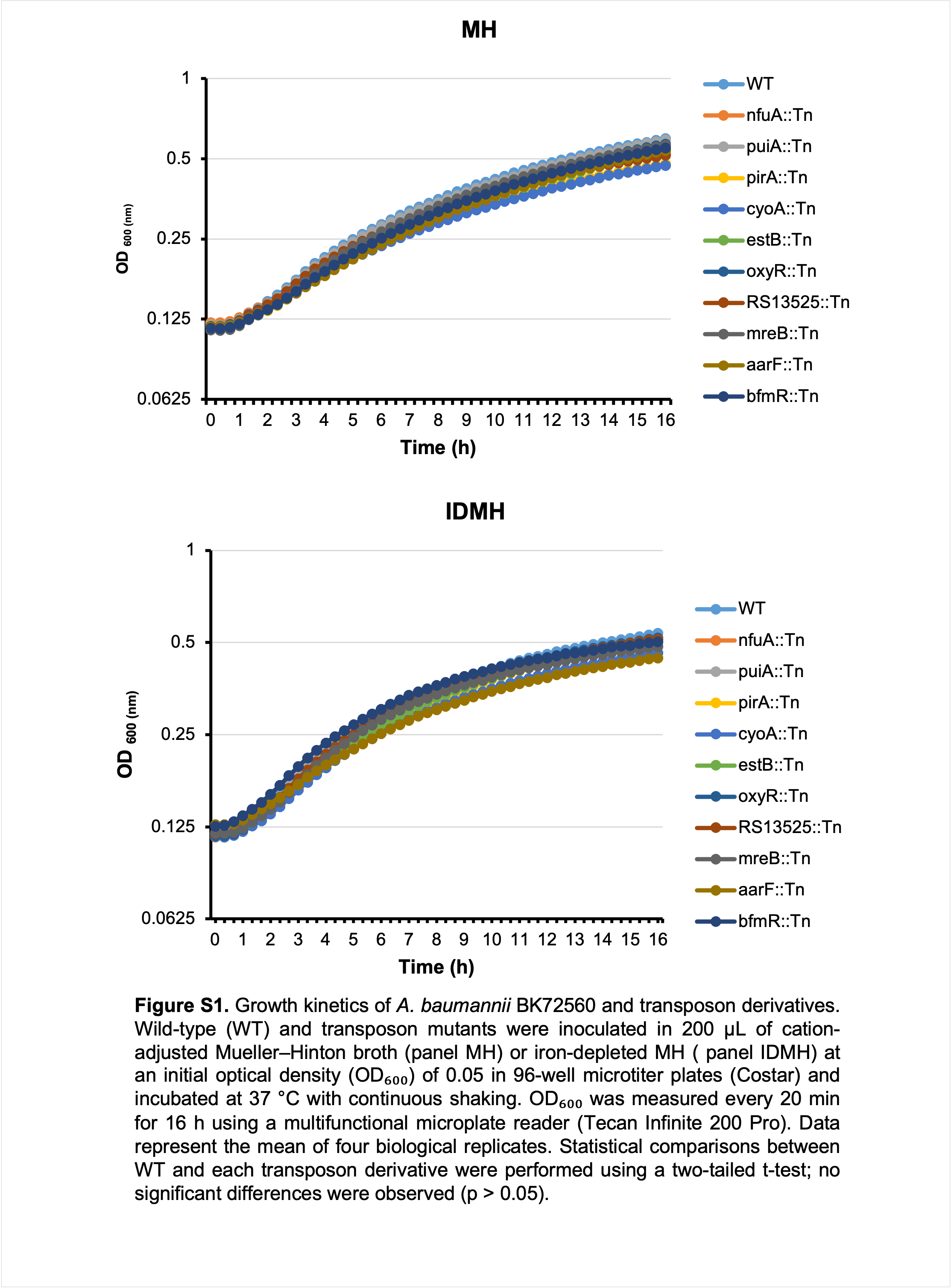

Supplement: Figure S1 — Growth kinetics of A. baumannii BK72560 and transposon derivatives in cation-adjusted Mueller-Hinton broth (panel MH) or iron-depleted MH (panel IDMH). [file spectrum.03804-25-s0001.tiff]

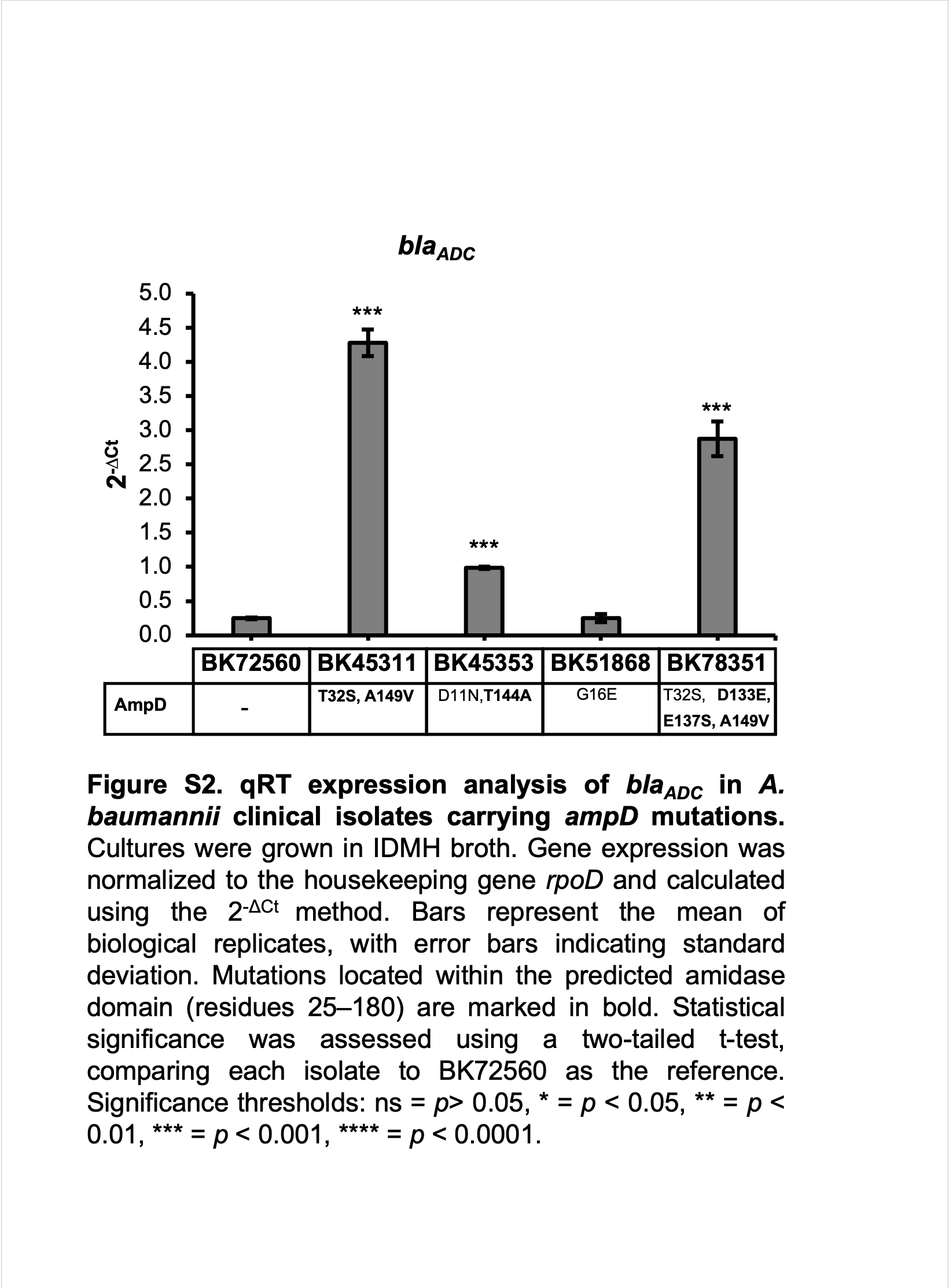

Supplement: Figure S2 — qRT expression analysis of blaADC in A. baumannii clinical isolates carrying ampD mutations. Cultures were grown in IDMH broth. Gene expression was normalized to the housekeeping gene rpoD and calculated using the 2-ΔCt method. [file spectrum.03804-25-s0002.tiff]
