# Supplementary material for: Genetic basis of cefiderocol resistance in Acinetobacter baumannii: insights from functional genomics and clinical isolates
Source: Microbiol Spectr. 2026 Feb 9;14(3):e03804-25. doi: 10.1128/spectrum.03804-25 (PMC12955420; doi:10.1128/spectrum.03804-25)
Supplement: Table S1 — Bacterial strains and plasmids used in this study. [file spectrum.03804-25-s0003.docx]

| **Strain** | **Genotype/Features** | **Strain Type** | **Reference** |
| --- | --- | --- | --- |
| BK72560 | Parental  *Acinetobacter baumanii* Sequence Type 2 | WT | This study |
| BK74024 | BK72560::TnHimar insertion in *pirA* | *pirA*::Tn mutant | This study |
| BK74990 | BK72560::TnHimar insertion 20bp upstream of *nfuA* | *nfuA*::Tn mutant | This study |
| BK74991 | BK72560::TnHimar insertion in *puiA* | *puiA*::Tn mutant | This study |
| BK74994 | BK72560::TnHimar insertion in RS13525 | RS13525::Tn mutant | This study |
| BK74996 | BK72560::TnHimar insertion in *oxyR* | *oxyR*::Tn mutant | This study |
| BK74998 | BK72560::TnHimar insertion in *estB* | *estB*::Tn mutant | This study |
| BK75001 | BK72560::TnHimar insertion in *aarF* | *aarF*::Tn mutant | This study |
| BK75004 | BK72560::TnHimar insertion in *bfmR* | *bfmR*::Tn mutant | This study |
| BK75006 | BK72560::TnHimar insertion in *cyoA* | *cyoA*::Tn mutant | This study |
| BK75007 | BK72560::TnHimar insertion in *mreB* | *mreB*::Tn mutant | This study |
| BK45355 | *Acinetobacter baumanii* Sequence Type 2 | CFDC- (S) isolate * | This study |
| BK51868 | *Acinetobacter baumanii* Sequence Type 2 | CFDC- (I) isolate | This study |
| BK45311 | *Acinetobacter baumanii* Sequence Type 2 | CFDC- (R) isolate | This study |
| BK45353 | *Acinetobacter baumanii* Sequence Type 2 | CFDC- (R) isolate | This study |
| BK74166 | *Acinetobacter baumanii* Sequence Type 2 | CFDC- (R) isolate | This study |
| BK78055 | *Acinetobacter baumanii* Sequence Type 2 | CFDC- (R) isolate | This study |
| BK78351 | *Acinetobacter baumanii* Sequence Type 2 | CFDC- (R) isolate | This study |

**Table S1. Bacterial Strains and Plasmids Used in This Study**

| **Plasmid** |  |  |
| --- | --- | --- |
| pSAMtac-1 | Tn Himar delivery plasmid, R6K ori | (21) |
| pMA-apra | *apmR*, *araB*p expression vector | (24) |
| pKR-AB-1 | pMA-apra derivatives: *rifR* *araB*p expression vector | This study |
| pKR-AB-2 | *araBp*-RS14160 (*pirA*) in pKR-AB-1 | This study |
| pKR-AB-3 | *araBp*-RS16540 (*puiA*) in pKR-AB-1 | This study |
| pKR-AB-4 | *araBp*-RS14165 (*nfuA*) in pKR-AB-1 | This study |
| pKR-AB-5 | *araBp*-RS14100 (*estB*)- RS14105 (*oxyR*) in pKR-AB-1 | This study |
| pKR-AB-6 | *araBp*-RS15450 (*bfmR*)- RS15455 (*bfmS*) in pKR-AB-1 | This study |
| pKR-AB-7 | *araBp*-RS07580 (*cyoA*) in pKR-AB-1 | This study |
| pKR-AB-8 | *araBp*-RS07705 (*aarF*) in pKR-AB-1 | This study |
| pKR-AB-9 | *araBp*-RS03530 (*mreB*) in pKR-AB-1 | This study |
| pKR-AB-10 | *araBp*-RS13525 in pKR-AB-1 | This study |

* Cefiderocol susceptibility categories (I, intermediate; R, resistant) were assigned according to (CLSI) 2023 breakpoints.
