# Supplementary material for: Genetic basis of cefiderocol resistance in Acinetobacter baumannii: insights from functional genomics and clinical isolates
Source: Microbiol Spectr. 2026 Feb 9;14(3):e03804-25. doi: 10.1128/spectrum.03804-25 (PMC12955420; doi:10.1128/spectrum.03804-25)
Supplement: Table S2 — Primers used in this study for arbitrary PCR and cloning. [file spectrum.03804-25-s0004.docx]

**Table S2. Primers Used in This Study for Arbitrary PCR and Cloning**

| **Primer Name** | **Sequence 5’-3’** | **Purpose** |
| --- | --- | --- |
| TnApr-F1 | ACGCTACGGAAGGAGCTGTG | Round 1 arbitrary PCR |
| ARB1 | GGCCACGCGTCGACTAGTAC(N_10)_GATAT |  |
| ARB1B | GGCCACGCGTCGACTAGTAC(N_10_)TGCGG |  |
| ARB1C | GGCCACGCGTCGACTAGTAC(N_10_)TCCGG |  |
| ARB6 | GGCCACGCGTCGACTAGTAC(N_10_)AGCGG |  |
| TnApr-F2 | TCCCCAAGGTTGAGAAGCTGA | Round 2 arbitrary PCR |
| ARB2 | GGCCACGCGTCGACTAGTAC |  |
| pMA-ara-F | CTGACGATCGAAACGTGGCCAATATGGACA | Construction pKRAB1 |
| pMA-ara-R | TGACGCGGCCGCTTGGTAACGAATCAGACAATTGAC |  |
| *rifR*-F | ACGTGCGGCCGCGGAACCCCTATTTGTTTATTTTTC |  |
| *rifR*-F | ACGTCGATCGAACTTGGTCTGACAGCTAGT |  |
| *pirA*-XbaI-F | AGTCTCTAGAAAAGAACACATTCCTGAAC | *pirA*::Tn complementation  (pKRAB2) |
| *pirA*-PstI-R | AGTCCTGCAGGCCCGTGTGTACTTAGAGAAG |  |
| *puiA*-BamHI-F | GATCAGGATCCCAATTAACACAAACTGTACATATAAATAAG | *puiA*::Tn complementation  (pKRAB3) |
| *puiA*-PstI-R | AGTCCTGCAGATAGCCTCTAACAAGAGAGGC |  |
| *nfuA*-PstI-F | AGTCCTGCAGCCTGTTGTGTATAAATCCGAC | *nfuA*::Tn complementation  (pKRAB4) |
| *nfuA*-XbaI-R | AGTCTCTAGACTCATTTTCAGTTTCGCATTGTAAC |  |
| *estB*-BamHI-F | GATCAGGATCCAGATCTTATTCCTGCCCAAGC | *estB*::Tn and *oxyR*::Tn  complementation (pKRAB5) |
| *oxyR*-XbaI-R | AGTCTCTAGAGATAGCTTGGATTTGAGATCGG |  |
| *bfmR*-XbaI-F | AGTCTCTAGAGGGTTGTCATGTATCAGTTTGGTG | *bfmR*::Tn complementation  (pKRAB6) |
| *bfmS*-EcoRI-R | AGTCGAATTCATGCACTCAATAGCCTGAGAAGC |  |
| *cyoA*-BamHI-F | GATCAGGATCCACTAGCGTTCTCAATCATGCGACAC | *cyoA*::Tn complementation  (pKRAB7) |
| *cyoA*-EcoRI-R | AGTCGAATTCGTCGGGATAGAGTCCCAACCC |  |
| *aarF*-BamHI-F | GATCAGGATCCACCATTCAGTCTGAGGGAAC | *aarF*::Tn complementation  (pKRAB8) |
| *aarF*-XbaI-R | AGTCTCTAGAGAATGACACGGAATCATTCTAGTC |  |
| *mreB*-BamHI-F | GATCAGGATCCGCGATTGACTTGTTCAAATC | *mreB*::Tn complementation  (pKRAB9) |
| *mreB*-HindIII-R | AGTCAAGCTTGAAAAAATATTCGGTTGCACC |  |
| RS13525-EcoRI-F | AGTCGAATTCGCCGATTCATTAAACAAGGC | RS13525::Tn complementation (pKRAB10) |
| RS13525-PstI-R | AGTCCTGCAGGCAGTAAAGCTTGAGCTAAGAT |  |
