# Supplementary material for: Genetic basis of cefiderocol resistance in Acinetobacter baumannii: insights from functional genomics and clinical isolates
Source: Microbiol Spectr. 2026 Feb 9;14(3):e03804-25. doi: 10.1128/spectrum.03804-25 (PMC12955420; doi:10.1128/spectrum.03804-25)
Supplement: Table S3 — Primers used for gene expression quantification by qRT-PCR. [file spectrum.03804-25-s0005.docx]

**Table S3. Primers Used for Gene Expression Quantification by qRT-PCR**

| **Locus Tag** | **Gene name** | **Description** | **Forward** | **Reverse** |
| --- | --- | --- | --- | --- |
| RS03025 | *btub3* | TBDT* | GAACACTGCCTCAACTGGTG | AGATCTGCGCCTACAATCGA |
| RS10605 | *bfnH* | TBDT | AGACGTGGCGAATAAATGGC | AATGCGGCTAATTCACGGTC |
| RS18465 |  | TBDT | TTAAAAGGACCAATGGCGGC | GCGTTCACTGCTGACATGAT |
| RS03255 | *yncD* | TBDT | GGAAATTCATCAGGCGGGAC | TTTCTGCACCGCTATGTTCG |
| RS00710 | *yddB* | TBDT | GGCACAACTTACGGCGTATT | GTTGCCATACCAAGCCAACA |
| RS08070 | *fauA* | TBDT | AGACCCGGTTGAAGGAAACA | TTCACTCGTCGCACCTTTTG |
| RS05745 | *bauA* | TBDT | AAATCAGCAACCACGCCTG | ATGTATGCGCAAATGTCCCC |
| RS16540 | *piuA* | TBDT | CTGGCGCTGTTTACTCTGAC | CTAGAACTGCACTACGGCCT |
| RS00790 | *fhuA* | TBDT | AAACAGGTTGAGGTCGGGAT | CCGCGAGTTAACTGTTCACC |
| RS13745 | *fecA* | TBDT | GTCCGAGCAACAAACCACTT | TGGTCACGGTAACTATCCCC |
| RS14160 | *pirA* | TBDT | AAAAGCCTGCTGAACCTGTG | ACCTGGCATACGACGTACAT |
| RS08765 |  | TBDT | TGGTTACCGTGATCAGGCTT | AGTGTCAACATCCCCAACCT |
| RS10225 |  | TBDT | GCTTTGCTGCCCATCTGAAT | CCCTGGCGATCGTTACAAAG |
| RS10520 |  | TBDT | TGGGGTGGACAAGAACATCA | AGATTGCGGTGTCCAACTTG |
| RS05870 |  | TBDT | CTGGAGCTTTGGTGATTGGG | CCCGTGTCATCTCGTTTCAC |
| RS08035 |  | TBDT | CTCAAGCCGAACCATTACCG | GAAGCGCCACCACCATAAAT |
| RS14495 |  | TBDT | ACTGAGTTTCCACGGTTTGC | TGCCGACAAACTCAACTGTG |
| RS18100 |  | TBDT | GCTTGCTGTCTCTCATTCGG | ATCGGTTGCTTTGGATCTGC |
| RS05830 | *ampC^33^* | class C β-lactamase | TGATAGCGACCCTGATGTGT | CAGCCGATTCGAGTTAACCG |
| RS05830 | *ampC^143^* | class C β-lactamase | ACCGGTAGTTGAGCCAGTTT | CCAGCAGATATTCAACGGGC |
| RS05830 | *ampC^212^* | class C β-lactamase | TGATAGCGACCCTGATGTGT | CAGCCGATTCGAGTTAACCC |
| RS05830 | *ampC^30^* | class C β-lactamase | CCAGCAGATATTCAACGGGC | ACCGGTAGTTGAGCCAGTTT |
| RS05830 | *ampC^293^* | class C β-lactamase | TGATAGCGACCCTGATGTGT | CAGCCGATTCGAGTTAACCC |
| RS04230 | *rpoD* | RNA polymerase σ factor | CGACTTATGCGACTTGGTGG | GTAGGTTCACGGCCCATTTC |

* TBDT, TonB-dependent transporter
